# Polyploidization increases meiotic recombination frequency in *Arabidopsis*

**DOI:** 10.1186/1741-7007-9-24

**Published:** 2011-04-21

**Authors:** Ales Pecinka, Wei Fang, Marc Rehmsmeier, Avraham A Levy, Ortrun Mittelsten Scheid

**Affiliations:** 1 Gregor Mendel Institute of Molecular Plant Biology, 1030 Vienna, Austria; 2 Max Planck Institute for Plant Breeding Research, Cologne, Germany; 3 Northwest A & F University, Shaanxi, P.R. China; 4 Uni Computing, Bergen, Norway; 5 Department of Plant Sciences, Weizmann Institute of Science, 76100 Rehovot, Israel

## Abstract

**Background:**

Polyploidization is the multiplication of the whole chromosome complement and has occurred frequently in vascular plants. Maintenance of stable polyploid state over generations requires special mechanisms to control pairing and distribution of more than two homologous chromosomes during meiosis. Since a minimal number of crossover events is essential for correct chromosome segregation, we investigated whether polyploidy has an influence on the frequency of meiotic recombination.

**Results:**

Using two genetically linked transgenes providing seed-specific fluorescence, we compared a high number of progeny from diploid and tetraploid *Arabidopsis *plants. We show that rates of meiotic recombination in reciprocal crosses of genetically identical diploid and autotetraploid *Arabidopsis *plants were significantly higher in tetraploids compared to diploids. Although male and female gametogenesis differ substantially in meiotic recombination frequency, both rates were equally increased in tetraploids. To investigate whether multivalent formation in autotetraploids was responsible for the increased recombination rates, we also performed corresponding experiments with allotetraploid plants showing strict bivalent pairing. We found similarly increased rates in auto- and allotetraploids, suggesting that the ploidy effect is independent of chromosome pairing configurations.

**Conclusions:**

The evolutionary success of polyploid plants in nature and under domestication has been attributed to buffering of mutations and sub- and neo-functionalization of duplicated genes. Should the data described here be representative for polyploid plants, enhanced meiotic recombination, and the resulting rapid creation of genetic diversity, could have also contributed to their prevalence.

## Background

Genome research, cytology, taxonomy, and archaeobotany provide evidence that most higher plants have polyploid ancestors [1]. Polyploidy is also frequent in extant species, including many crops [2]. The benefit of polyploidy in natural and anthropogenic evolution has been attributed to a series of factors accelerating evolution, such as mutation buffering, dosage effects, increased allelic diversity and heterozygosity, and sub- or neo-functionalization of duplicated genes and resulting phenotypic variation (reviewed in [3]). However, polyploidization can also destabilize gene regulation networks, genome organization, and chromosome segregation (reviewed in [3]). A critical stage in the evolution of polyploids is stabilization of meiosis, especially the assortment of homologous chromosomes (homologs) and their equal distribution in the first meiotic division. A lack of crossing over between homologs leads to defective chromosome segregation in most species [4,5]. In autopolyploids, where a single genome is duplicated, homologs can form multivalents with the risk of unequal segregation. Meiotic chromosomes in allopolyploids, where two or more different genomes are combined, usually show a diploid-like behavior. However, in some cases, pairing between homoeologous chromosomes (homoeologs) can occur, causing genetic instability [6,7]. Correct pairing is a prerequisite for efficient meiotic recombination. In addition to being a major mechanism for the creation of new combinations of parental traits, recombination is also required for subsequent balanced chromosome segregation. Although several studies have documented altered pairing behavior in auto- and allopolyploids [6-8], a systematic comparison of meiotic recombination frequencies (MRFs) between diploids and auto- and allopolyploids has been lacking. We demonstrate that rates of meiotic recombination between two genetically linked markers in *Arabidopsis *are significantly higher in tetraploids than in diploids.

## Results

### Determining meiotic recombination frequency in diploids

MRF can be defined as the probability that two paired segments of non-sister chromatids recombine. To determine MRF in diploids and tetraploids, we used an MRF assay with visible markers previously established [9]. *Arabidopsis thaliana *line 3A [Col3-4/20 sensu 9] contains transgenic inserts coding for Green and Red Fluorescent Proteins (GFP and RFP), driven by a seed-specific promoter. Both inserts are located on the top arm of chromosome 3, GFP distal and RFP proximal, with a physical distance of 5.1 Mb and a genetic distance of 16.3 cM [9]. Seeds with both markers appear yellow upon UV excitation; the occurrence of seeds with exclusively green or exclusively red fluorescence indicates a meiotic recombination event between the markers. From the relative frequencies of these events, MRFs can be estimated [9]. Both markers were stably expressed over several generations [9], and plants homozygous for both markers exclusively produced seeds with yellow fluorescence also during our experiments (n = 3177, progeny from three individual plants).

**Figure 1:**
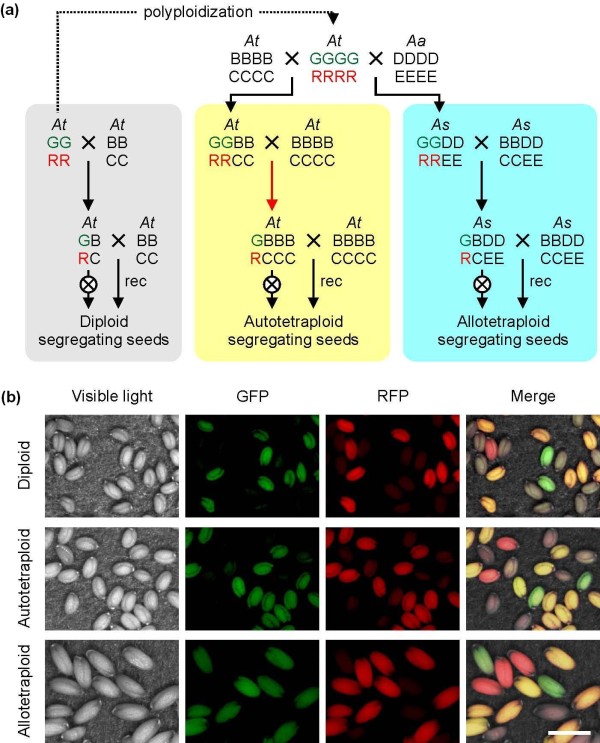
**The Meiotic Recombination Frequency (MRF) assay**. (A) Crossing scheme: *At *- *Arabidopsis thaliana*, *Aa *- *Arabidopsis arenosa*, *As *- *Arabidopsis suecica*. G = Green Fluorescent Protein (GFP) and R = Red Fluorescent Protein (RFP) are transgenic markers on the upper arm of chromosome 3 in the *Arabidopsis thaliana *genome. MRF assay in diploid (grey), autotetraploid (yellow) and allotetraploid (blue) background. B and C denote the corresponding transgene-free location, D and E indicate the homoeologous positions in the *Arabidopsis arenosa *genome. The copy number is shown as number of letters for each position. Red arrow; yellow seeds carrying a single copy of each marker were selected; crossed circle - self-pollination; rec - reciprocal cross. (B) Examples for seed phenotypes obtained from mother plants with a single copy of the meiotic tester chromosome pollinated by wild type (female recombination). Scale bar, 1 mm.

To analyze diploid MRF, line 3A, homozygous for both marker genes, was crossed with a non-transgenic wild type to produce diploid F_1 _plants with only one non-recombined chromosome (Figure 1, grey panel). After selfing, the progeny plants indicated an MRF of 15.4% (Table 1 and Additional File 1), not significantly different (*P *> 0.1, unpaired *t*-test) from the previously published value of 16.3% [9]. Backcrosses of the heterozygous F_1 _plants in reciprocal configuration indicated a female MRF of 7.4% and a male MRF of 20.2% (Table 1). The significant (*P *< 0.0001) 2.7-fold difference is in agreement with published genetic and cytological data [10,11].

**Table 1:** Meiotic recombination frequencies (MRF) in diploid, autotetraploid and allotetraploid Arabidopsis^1^.

Ploidy (species)	Meiosis^2^	Plants	Seed fluorescence				Seeds total	MRF (%)	S.D.^4 ^(%)
						
			Green-only	Red-only	Yellow^3^	None			
Diploid *A. thaliana*	Female	6	66	71	830	894	1861	7.4	1.9
	Selfed	3	322	333	2805	791	4251	15.4	0.9
	Male	3	147	143	561	582	1433	20.2	0.3
									
Autotetraploid *A. thaliana*	Female	10	264	317	1587	1703	3871	15.0	3.2
	Selfed	10	1868	2216	12707	3098	19889	20.5	1.1
	Male	9	506	492	1227	1345	3570	28.0	3.0
									
Allotetraploid *A. suecica*	Female	5	181	214	1348	1305	3048	13.0	2.5
	Selfed	5	275	298	1484	320	2377	24.1	1.8
	Male	5	598	599	1412	1410	4019	29.8	3.1

^1 ^Detailed values for individual plants are given in Additional Files 1,2 and 3

^2 ^Transmission of the meiotic recombination tester through maternal (female), paternal (male) or both gametes (selfed) determined by reciprocal crosses (female, male) or self-pollination.

^3 ^Seeds showing both red and green fluorescence.

^4 ^S.D. - standard deviation

### Meiotic recombination frequency in autotetraploids

To determine MRFs in autotetraploids, we identified plants with a stable and complete tetraploid chromosome set of 4 × = 20 among the progeny of a colchicine-treated diploid, homozygous line 3A. Seeds from these plants (n = 3652, progeny from five individual plants, three generations after polyploidization) exclusively showed yellow fluorescence, indicating stable expression of both markers after polyploidization. The tetraploid derivatives, with four copies of the marker chromosome, were backcrossed to non-transgenic autotetraploid *Arabidopsis thaliana *(Figure 1, yellow panel). This results in plants carrying two copies of the transgenic chromosome 3. To exclude a dependence of MRF on the copy number and to strictly compare with diploids having a single marker chromosome, we backcrossed the double-copy lines once more to wild-type plants (Figure 1). The intensity of red and green fluorescence depends on the dosage of the marker genes. With the double-copy F1 plants as reference, plants with one gene copy each were selected and used for the final test crosses. We have taken into account that markers could have been separated by recombination already during the backcross, but nevertheless be transmitted through the same gamete. However, in this case, they would segregate like uncoupled markers in the selfed progeny, and we have retrospectively eliminated such plants from the analysis. The single copy plants were backcrossed once more to the tetraploid wild type, again in reciprocal orientation, or allowed to self-pollinate. Again, female and male MRFs were significantly different from each other (*P *< 0.0000001), but more importantly, single-copy autotetraploid MRFs were significantly higher in comparison to diploid MRFs (selfing MRF = 20.5%, *P *< 0.0001; female MRF = 15.0%, *P *< 0.001; male MRF = 28.0%, *P *< 0.001, Table 1 and Additional File 2).

We observed a slight tendency towards more red-only than green-only seeds [see Additional File 2], and potentially, this could have been caused by silencing of the GFP marker gene in the polyploids, as seen for other transgenes [12-14]. Therefore, we tested for the presence of the GFP marker in the genomic DNA of 12 plants grown from red-only seeds and derived from several individual parents, using PCR. All were negative, indicating that the green marker was indeed lost by recombination. Some bias towards red-only seeds could be the result of double reduction, a special type of meiotic recombination in the case of multivalent formation in autotetraploids in which sister chromatids end up in the same gamete [15]. Its frequency depends on the distance between marker and centromere and is therefore different between proximal and distal positions. However, double reduction cannot serve as an explanation in allopolyploids. Potentially, a bias can also originate from unequal fitness and transmission of gametes with a specific recombined chromosome, but this is rather unlikely in the case of the autopolyploids.

### Meiotic recombination frequency in allotetraploids

**Figure 2:**
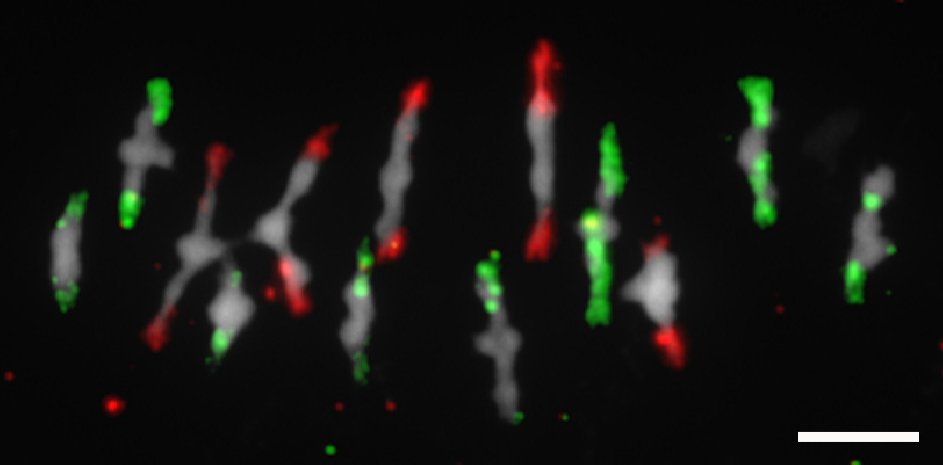
**Strict bivalent formation in allotetraploids**. Chromosomes of synthetic *Arabidopsis suecica *at meiotic metaphase I stage were hybridized with species-specific centromeric repeats of *Arabidopsis thaliana *(red) and *Arabidopsis arenosa *(green) and show pairing exclusively between homologous chromosomes of the respective genomes. Scale bar, 5 μm.

Increased MRF in autotetraploids could potentially be connected with multivalent formation, the major distinction of autotetraploid meiosis (described above), due to the homology of four chromosomes. Therefore, we generated an equivalent assay system for scoring MRF in tetraploids with only bivalent formation. *Arabidopsis arenosa *is a natural autotetraploid and close relative of *Arabidopsis thaliana*. Crosses between tetraploid *Arabidopsis thaliana *and *Arabidopsis arenosa *give rise to fertile allopolyploid hybrids that resemble *Arabidopsis suecica*, a species originating from an ancient hybridization event between these species [16]. We crossed the homozygous tetraploid line 3A with non-transgenic tetraploid *Arabidopsis arenosa *(4 × = 32) (Figure 1, blue panel) and identified plants with stable and full parental chromosome sets of 4 × = 26. Chromosomes derived from the two parental genomes in *Arabidopsis suecica *and in newly established hybrids were shown to pair in a diploid-like manner [6]. To verify the strict exclusion of multivalent formation or pairing between non-homologous chromosomes, we performed a cytological analysis of the resulting hybrids during the transition from meiotic metaphase I to anaphase I. Chromosomes from different parental origin were distinguished by fluorescence *in situ *hybridization with differentially labeled species-specific centromeric repeat probes. Among 55 metaphases analyzed, *Arabidopsis thaliana *and *Arabidopsis arenosa *chromosomes were found as homologous bivalents in 99.3% and 98.0%, respectively (Figure 2), with the few remaining chromosome pairs already separated due to the onset of anaphase I. No obvious case of pairing between homoeologs was detected. Thus, the newly synthesized hybrids, containing the meiotic tester chromosome, also showed a strictly diploid-like behavior of meiotic chromosomes. Subsequent crosses of the hybrids (this time to *Arabidopsis suecica*), identification of plants with one non-recombined chromosome, and the final cross in reciprocal orientation and with self-pollination, were performed as for autotetraploids (Figure 1, blue panel). Selfing, female, and male MRFs (24.1%, 13.0%, and 29.8%, respectively, Table 1 and Additional File 3) were again significantly higher than those in diploids (*P *< 0.001, *P *< 0.01, and *P *< 0.01, respectively) and in the same range as those in autotetraploids. These results suggest that the increase of MRF in tetraploids is independent of the formation of multivalents and pairing-partner switches.

## Discussion

We have demonstrated that two genetically linked markers are more often separated by meiotic recombination in tetraploid than in diploid *Arabidopsis*. The comparison of meiotic recombination rates between diploids and isogenic autotetraploids allowed us to attribute the higher MRF entirely to the increased ploidy, excluding any effect of divergent genetic background. In the newly synthesized allotetraploid *Arabidopsis suecica *hybrids, genetic differences could, in principle, be responsible for the observed higher rates. However, the MRF changes in the newly synthesized *Arabidopsis suecica *hybrids are similar to those observed in autotetraploids in both direction and degree, supporting the notion that the polyploid condition *per se *is responsible for higher MRFs. The seed-specific expression of the originally linked fluorescence marker genes, and the possibility of generating many seeds upon selfing and reciprocal backcrossing, enabled us to perform the analysis with large numbers, thereby providing high statistical power. Achieving comparable confidence with other endogenous marker types would be extremely intricate and costly. Although the assay used here is restricted to quantifying recombination rates between a specific marker pair, it is unlikely that the segment defined by this pair represents an exceptional region of the genome. The markers encompass 5 Mb, approximately 20% of chromosome 3, outside of the centromere [17] and can be considered as typical of the euchromatin fraction of the *Arabidopsis *genome. Moreover, a region of this size is expected to include several recombinational hot and cold spots and thereby balance most of the local fluctuations in MRFs that have been reported across the *Arabidopsis *chromosomes [18]. In addition, the two markers in the diploid tester line 3A yield stable recombination rates over several generations in agreement with genetic maps established with non-transgenic markers. Another limitation of the assay at present is its restriction to recently generated hybrids. However, inserting transgenic markers into established natural polyploids and/or production of advanced polyploid generations from synthetic lines will take a long time. To investigate how other genomic regions are affected, and whether the increased MRFs will persist in later generations of tetraploids remains to be investigated. Nevertheless, even a regional and/or transient MRF increase in new polyploids is potentially meaningful for generating genetic diversity. Recent observations of increased crossover frequency and extended linkage maps in allotriploid and allotetraploid *Brassica *hybrids [8] suggest that our results could be valid beyond the study described here.

What is the possible mechanism for the influence of polyploidy on MRF? The endonuclease SPO11 creates many more double strand breaks upon the onset of meiosis than are processed into subsequent crossover events (reviewed in [19]). Therefore, increased recombination frequencies are likely determined later during strand exchange and DNA repair. Chromosomes might be less condensed in tetraploids, thus rendering the chromatids more accessible for end processing and strand invasion and explaining an increased MRF. Differences in recombination frequencies between male and female meiosis were correlated with different lengths of the synaptonemal complex (SC) as revealed by immunostaining with antibodies against the SC-specific protein ZYP1 [10]. We measured the distance between the markers with fluorescence *in situ *hybridization (FISH) but the data did not indicate length differences between pachytene preparations from diploid and autotetraploid plants. Nevertheless, chromatin is specifically reorganized preceding zygotene in maize [20] and recombinational hotspots in mouse have lower nucleosome occupancy than less recombinogenic regions [21]. Therefore, differences in polyploid chromatin configuration, or in epigenetic features, are conceivable. Other tetraploid-specific factors could include differences in pairing initiation, delayed or enhanced progression through the meiotic cell cycle stages, altered dynamics of chromosome pairing [22], altered crossover interference [10] or different response to the genetic control of chromosome pairing [23,24]. In *Caenorhabditis elegans*, single unpaired chromosomes delay progression through meiosis and increase the number of crossovers [25]. This suggests a pairing checkpoint control that might also be sensitive to different efficiency of pairing in tetraploids. Further, pairing and recombination take place prior to nuclear envelope breakdown in early metaphase [26], and the ratio between nuclear volume and nuclear envelope area could constitute another difference between diploids and tetraploids. Finally, any component of the homologous recombination machinery could vary in activity between diploids and tetraploids, through differences in gene expression, translation efficiency, or protein modification.

## Conclusions

Evidence for genetic and epigenetic changes in polyploids and their potential evolutionary benefit have been discussed repeatedly (for review see [27,28]). Increased MRFs could boost the amount of genetic diversity in polyploids, thereby enhancing their adaptation potential in challenging natural environments. Furthermore, enhanced recombination could help stabilize the chromosome complement and segregation during complex meiosis, or enhance the formation of bivalents in allotetraploids. Polyploidy-enhanced MRF might thus have a dual impact, enhancing the generation of new diversity, while at the same time, promoting genome stability through proper chromosome segregation.

Generating new combinations of genetic material has also been a major issue in plant breeding. Introgression of interesting traits into crop plants is nowadays mainly achieved by marker-assisted analysis of segregating populations after hybridization. This approach can be limited by a low frequency of meiotic recombination events in the neighboring genomic region. Any enhancement of MRF could be favorable for saving time, labor, and costs, especially in species with long generation times. It is conceivable that enhanced recombination rates in polyploid plants have led to a rapid diversification and optimization during domestication, once polyploid derivatives from diploid progenitors were utilized, as for example, in wheat [29]. While the influence of polyploidy on the frequency of meiotic recombination in other species awaits confirmation, genetic approaches in *Arabidopsis *should help to understand the connection. This may provide a means to enhance genetic exchange in hybrids or regulate meiotic recombination otherwise.

## Methods

### Plant material and growth conditions

*Arabidopsis thaliana *Col-0 line 3A [Col3-4/20, 9] harbors GFP and RFP transgenes on the top arm of chromosome 3, at nucleotide positions 256,516 and 5,361,637, respectively. All marker genes were homozygous in the diploid starting material. Seeds of autotetraploid *Arabidopsis thaliana*, autotetraploid *Arabidopsis arenosa, *and synthetic allotetraploid *Arabidopsis suecica *were obtained from L. Comai (University of California, Davis, USA). All plants were grown at 22°C under long-day (16 hour light: 8 hour dark) conditions.

### Polyploidization

Two week-old *in vitro *grown plants of line 3A were flooded with 0.1% colchicine dissolved in tap water for two hours, rinsed extensively with tap water, transferred to soil, and grown to set seeds. Nuclear DNA content of their progeny plants was analyzed by ploidy analyzer PAII (Partec, Münster, Germany). Tetraploid candidates were selected and the karyotype confirmed by counting mitotic chromosomes.

### Preparation of chromosomes and fluorescence *in situ *hybridization (FISH)

Meiotic or mitotic chromosomes were prepared from young inflorescences as described [30]. FISH probes specific for centromeric repeats were amplified from *Arabidopsis thaliana *and *Arabidopsis arenosa *genomic DNA and directly labeled with biotin-dUTP and digoxigenin-dUTP, respectively, during PCR. Primers for amplification of *Arabidopsis thaliana *repeats were CGT TCA ACG GAC CGG ATG ACA AA (180 bp-F) and GAC TTC AGC TAC ACT ATT AAA GT (180 bp-R), those for *Arabidopsis arenosa *repeats AGT CTT TGG CTT TGT GTC TT (ALU) and TGG ACT TTG GCT ACA CCA TG (ALR). Slide pre-treatment, hybridization and detection were performed as described [31].

### Evaluation of seed fluorescence and microscopy

Mature, dry seeds were photographed using a MZ 16 FA stereo-microscope equipped with a set of GFP- and RFP-specific filters and a DFC 300 FX camera (all from Leica, Wetzlar, Germany). Microscopic analysis of chromosomes was done with an AxioImager.Z1 (Zeiss, Jena, Germany) equipped with a Spot Pursuit camera (Diagnostic Instruments, Sterling Heights, MI, USA). Images were assembled and analyzed in Photoshop (Adobe Systems, San Jose, CA, USA).

## Authors' contributions

AP, AAL and OMS designed the research; AP generated tetraploid plants and performed the cytological analysis; AP and WF performed crosses and evaluated seed fluorescence; AP, WF, MR, AAL and OMS analyzed the data; AP, MR, AAL and OMS wrote the manuscript.

## Supplementary Material

Additional file 1 **Additional Table 1**. Click here for file

Additional file 2 **Additional Table 2**. Click here for file

Additional file 3 **Additional Table 3**. Click here for file
