# Correction: Polyploidization increases meiotic recombination frequency in *Arabidopsis*

**DOI:** 10.1186/1741-7007-10-33

**Published:** 2012-04-18

**Authors:** Ales Pecinka, Wei Fang, Marc Rehmsmeier, Avraham A Levy, Ortrun Mittelsten Scheid

**Affiliations:** 1 Gregor Mendel Institute of Molecular Plant Biology, 1030 Vienna, Austria; 2 Max Planck Institute for Plant Breeding Research, Cologne, Germany; 3 Northwest A & F University, Shaanxi, P.R. China; 4 Uni Computing, Bergen, Norway; 5 Department of Plant Sciences, Weizmann Institute of Science, 76100 Rehovot, Israel

## Correction

The authors noted that three values in Table 1 need corrections [1]. The MRF for self-pollinated diploids should be corrected from 15.4% to 16.8%, for self-pollinated autotetraploids from 20.5% to 23.2%, and for self-pollinated allotetraploids from 24.1% to 28.0%, applying the correct formula for genotype combinations upon self-pollination given in reference nine. Similar corrections need to be done to Additional Files 1, 2 and 3. See also Rehmsmeier 2012 [2]. The changes do not affect any of the conclusions presented in the original manuscript. We apologize for any inconvenience caused by this error.

**Table 1:** Meiotic recombination frequencies (MRF) in diploid, autotetraploid and allotetraploid Arabidopsis^1^

Ploidy	Meiosis^2^	Plants	Seed fluorescence				Seeds total	MRF (%)	S.D.^4 ^(%)
						
(species)			Green-only	Red-only	Yellow^3^	None			
Diploid	Female	6	66	71	830	894	1861	7.4	1.9
*A. thaliana*	Selfed	3	322	333	2805	791	4251	16.8	1.1
	Male	3	147	143	561	582	1433	20.2	0.3
Autotetraploid	Female	10	264	317	1587	1703	3871	15.0	3.2
*A. thaliana*	Selfed	10	1868	2216	12707	3098	19889	23.2	1.4
	Male	9	506	492	1227	1345	3570	28.0	3.0
Allotetraploid	Female	5	181	214	1348	1305	3048	13.0	2.5
*A. suecica*	Selfed	5	275	298	1484	320	2377	28.0	2.6
	Male	5	598	599	1412	1410	4019	29.8	3.1

^1 ^Detailed values for individual plants are given in Additional Files 1,2 and 3

^2 ^Transmission of the meiotic recombination tester through maternal (female), paternal (male) or both gametes (selfed) determined by reciprocal crosses (female, male) or self-pollination.

^3 ^Seeds showing both red and green fluorescence.

^4 ^S.D. - standard deviation, calculated from the individual crosses/self-pollinations in Additional Files 1, 2 and 3

## Supplementary Material

Additional file 1 **Additional Table 1**. Click here for file

Additional file 2 **Additional Table 2**. Click here for file

Additional file 3 **Additional Table 3**. Click here for file
