# Supplementary material for: Polyploidization increases meiotic recombination frequency in Arabidopsis
Source: BMC Biol. 2011 Apr 21;9:24. doi: 10.1186/1741-7007-9-24 (PMC3110136; doi:10.1186/1741-7007-9-24)
Supplement: Additional file 1 — Additional Table 1. [file 1741-7007-9-24-S1.PDF]

Additional File 1

Additional Table 1
Meiotic recombination frequencies (MRF) in diploid *A. thaliana* with single copy meiotic tester

Meiosis[1]	Plant ID	Seed fluorescence				Seeds total	MRF (%)	S.D.[3] (%)
		Green-only	Red-only	Yellow[2]	None			
Female	#01	12	8	196	185	401	5.0	
	#02	12	16	107	134	269	10.4	
	#03	15	15	221	219	470	6.4	
	#04	3	7	50	49	109	9.2	
	#05	2	6	46	47	101	7.9	
	#07	22	19	210	260	511	8.0	
	**Total**	**66**	**71**	**830**	**894**	**1861**	**7.4**	**1.9**
Selfing	#01	183	179	1612	465	2439	14.8	
	#02	123	131	1025	298	1577	16.1	
	#03	16	23	168	28	235	16.6	
	**Total**	**322**	**333**	**2805**	**791**	**4251**	**15.4**	**0.9**
Male	#01	47	56	189	214	506	20.4	
	#02	74	60	264	261	659	20.3	
	#03	26	27	108	107	268	19.8	
	**Total**	**147**	**143**	**561**	**582**	**1433**	**20.2**	**0.3**

[1] Transmission of the meiotic recombination tester through maternal (female), paternal (male) or both gametes (selfed) determined by reciprocal crosses (female, male) or self-pollination

[2] Seeds showing both red and green fluorescence

[3] S.D. - standard deviation
