# Supplementary material for: Polyploidization increases meiotic recombination frequency in Arabidopsis
Source: BMC Biol. 2011 Apr 21;9:24. doi: 10.1186/1741-7007-9-24 (PMC3110136; doi:10.1186/1741-7007-9-24)
Supplement: Additional file 2 — Additional Table 2. [file 1741-7007-9-24-S2.PDF]

Additional File 2

Additional Table 2
Meiotic recombination frequencies (MRF) in autotetraploid *A. thaliana* with single copy meiotic tester

Meiosis[1]	Plant ID	Seed fluorescence				Seeds total	MRF (%)	S.D.[3] (%)
		Green-only	Red-only	Yellow[2]	None			
Female	#01	17	26	95	98	236	18.2	
	#02	23	32	95	104	254	21.7	
	#03	16	12	106	131	265	10.6	
	#04	28	26	126	183	363	14.9	
	#05	18	23	146	157	344	11.9	
	#06	29	42	237	225	533	13.3	
	#07	20	31	148	146	345	14.8	
	#08	52	47	231	246	576	17.2	
	#09	23	26	135	135	319	15.4	
	#10	38	52	268	278	636	14.2	
	**Total**	**264**	**317**	**1587**	**1703**	**3871**	**15.0**	**3.2**
Selfing	#01	97	118	749	188	1152	18.7	
	#02	140	186	933	230	1489	21.9	
	#03	185	197	1214	272	1868	20.4	
	#04	187	199	1298	324	2008	19.2	
	#05	173	246	1237	324	1980	21.2	
	#06	171	212	1221	297	1901	20.1	
	#07	126	149	925	210	1410	19.5	
	#08	232	266	1601	372	2471	20.2	
	#09	245	293	1668	407	2613	20.6	
	#10	312	350	1861	474	2997	22.1	
	**Total**	**1868**	**2216**	**12707**	**3098**	**19889**	**20.5**	**1.1**
Male	#01	32	21	48	64	165	32.1	
	#02	74	71	147	163	455	31.9	
	#03	34	50	118	84	286	29.4	
	#04	45	49	120	130	344	27.3	
	#05	73	75	218	224	590	25.1	
	#06	120	111	278	310	819	28.2	
	#07	49	41	84	121	295	30.5	
	#08	39	32	93	101	265	26.8	
	#09	40	42	121	148	351	23.4	
	**Total**	**506**	**492**	**1227**	**1345**	**3570**	**28.0**	**3.0**

[1] Transmission of the meiotic recombination tester through maternal (female), paternal (male) or both gametes (selfed) determined by reciprocal crosses (female, male) or self-pollination.

[2] Seeds showing both red and green fluorescence.

[3] S.D. - standard deviation
