# Supplementary material for: Polyploidization increases meiotic recombination frequency in Arabidopsis
Source: BMC Biol. 2011 Apr 21;9:24. doi: 10.1186/1741-7007-9-24 (PMC3110136; doi:10.1186/1741-7007-9-24)
Supplement: Additional file 3 — Additional Table 3. [file 1741-7007-9-24-S3.PDF]

Additional File 3

Additional Table 3
Meiotic recombination frequencies (MRF) in allotetraploid *A. suecica* with single copy meiotic tester

Meiosis[1]	Plant ID	Seed fluorescence				Seeds total	MRF (%)	S.D.[3] (%)
		Green-only	Red-only	Yellow[2]	None			
Female	#01	31	33	283	268	615	10.4	
	#02	45	53	268	304	670	14.6	
	#03	25	31	262	236	554	10.1	
	#06	49	44	263	240	596	15.6	
	#07	31	53	272	257	613	13.7	
	**Total**	**181**	**214**	**1348**	**1305**	**3048**	**13.0**	**2.5**
Selfing	#01	59	61	315	79	514	23.3	
	#02	35	44	207	52	338	23.4	
	#03	30	31	186	40	287	21.3	
	#06	65	68	320	53	506	26.3	
	#07	86	94	456	96	732	24.6	
	**Total**	**275**	**298**	**1484**	**320**	**2377**	**24.1**	**1.8**
Male	#01	121	117	227	256	721	33.0	
	#02	151	147	314	355	967	30.8	
	#03	53	67	211	156	487	24.6	
	#06	122	111	285	247	765	30.5	
	#07	151	157	375	396	1079	28.5	
	**Total**	**598**	**599**	**1412**	**1410**	**4019**	**29.8**	**3.1**

[1] Transmission of the meiotic recombination tester through maternal (female), paternal (male) or both gametes (selfed) determined by reciprocal crosses (female, male) or self-pollination

[2] Seeds showing both red and green fluorescence

[3] S.D. - standard deviation
