# Supplementary material for: Correction: Polyploidization increases meiotic recombination frequency in Arabidopsis
Source: BMC Biol. 2012 Apr 18;10:33. doi: 10.1186/1741-7007-10-33 (PMC3361494; doi:10.1186/1741-7007-10-33)
Supplement: Additional file 1 — Additional Table 1. [file 1741-7007-10-33-S1.DOC]

| **Additional Table 1** | | | | | | | | |
| --- | --- | --- | --- | --- | --- | --- | --- | --- |
| **Meiotic recombination frequencies (MRF) in diploid *A. thaliana* with single copy meiotic tester** | | | | | | | | |
| **Meiosis^1^** | **Plant ID** | **Seed fluorescence** | | | | **Seeds total** | **MRF (%)** | **S.D.^3^ (%)** |
|  |  | **Green-only** | **Red-only** | **Yellow^2^** | **None** |  |  |  |
| Female | #01 | 12 | 8 | 196 | 185 | 401 | 5.0 |  |
|  | #02 | 12 | 16 | 107 | 134 | 269 | 10.4 |  |
|  | #03 | 15 | 15 | 221 | 219 | 470 | 6.4 |  |
|  | #04 | 3 | 7 | 50 | 49 | 109 | 9.2 |  |
|  | #05 | 2 | 6 | 46 | 47 | 101 | 7.9 |  |
|  | #07 | 22 | 19 | 210 | 260 | 511 | 8.0 |  |
|  | **Total** | **66** | **71** | **830** | **894** | **1861** | **7.4** | **1.9** |
|  |  |  |  |  |  |  |  |  |
| Selfing | #01 | 183 | 179 | 1612 | 465 | 2439 | 16.1 |  |
|  | #02 | 123 | 131 | 1025 | 298 | 1577 | 17.7 |  |
|  | #03 | 16 | 23 | 168 | 28 | 235 | 18.3 |  |
|  | **Total** | **322** | **333** | **2805** | **791** | **4251** | **16.8** | **1.1** |
|  |  |  |  |  |  |  |  |  |
| Male | #01 | 47 | 56 | 189 | 214 | 506 | 20.4 |  |
|  | #02 | 74 | 60 | 264 | 261 | 659 | 20.3 |  |
|  | #03 | 26 | 27 | 108 | 107 | 268 | 19.8 |  |
|  | **Total** | **147** | **143** | **561** | **582** | **1433** | **20.2** | **0.3** |
| ^1^ Transmission of the meiotic recombination tester through maternal (female), paternal (male) or both gametes (selfed) determined by reciprocal crosses (female, male) or self-pollination | | | | | | | | |
|  |  |  |  |  |  |  |  |  |
| ^2^ Seeds showing both red and green fluorescence | | | | | | | | |
| ^3^ S.D. - standard deviation, calculated from the individual crosses/self-pollinations | | | | | | | | |
