# Supplementary material for: Correction: Polyploidization increases meiotic recombination frequency in Arabidopsis
Source: BMC Biol. 2012 Apr 18;10:33. doi: 10.1186/1741-7007-10-33 (PMC3361494; doi:10.1186/1741-7007-10-33)
Supplement: Additional file 2 — Additional Table 2. [file 1741-7007-10-33-S2.DOC]

| **Additional Table 2** | | | | | | | | |
| --- | --- | --- | --- | --- | --- | --- | --- | --- |
| **Meiotic recombination frequencies (MRF) in autotetraploid *A. thaliana* with single copy meiotic tester** | | | | | | | | |
| **Meiosis^1^** | **Plant ID** | **Seed fluorescence** | | | | **Seeds total** | **MRF (%)** | **S.D.^3^ (%)** |
|  |  | **Green-only** | **Red-only** | **Yellow^2^** | **None** |  |  |  |
| Female | #01 | 17 | 26 | 95 | 98 | 236 | 18.2 |  |
|  | #02 | 23 | 32 | 95 | 104 | 254 | 21.7 |  |
|  | #03 | 16 | 12 | 106 | 131 | 265 | 10.6 |  |
|  | #04 | 28 | 26 | 126 | 183 | 363 | 14.9 |  |
|  | #05 | 18 | 23 | 146 | 157 | 344 | 11.9 |  |
|  | #06 | 29 | 42 | 237 | 225 | 533 | 13.3 |  |
|  | #07 | 20 | 31 | 148 | 146 | 345 | 14.8 |  |
|  | #08 | 52 | 47 | 231 | 246 | 576 | 17.2 |  |
|  | #09 | 23 | 26 | 135 | 135 | 319 | 15.4 |  |
|  | #10 | 38 | 52 | 268 | 278 | 636 | 14.2 |  |
|  | **Total** | **264** | **317** | **1587** | **1703** | **3871** | **15.0** | **3.2** |
|  |  |  |  |  |  |  |  |  |
| Selfing | #01 | 97 | 118 | 749 | 188 | 1152 | 20.8 |  |
|  | #02 | 140 | 186 | 933 | 230 | 1489 | 25.0 |  |
|  | #03 | 185 | 197 | 1214 | 272 | 1868 | 23.1 |  |
|  | #04 | 187 | 199 | 1298 | 324 | 2008 | 21.5 |  |
|  | #05 | 173 | 246 | 1237 | 324 | 1980 | 24.1 |  |
|  | #06 | 171 | 212 | 1221 | 297 | 1901 | 22.7 |  |
|  | #07 | 126 | 149 | 925 | 210 | 1410 | 21.9 |  |
|  | #08 | 232 | 266 | 1601 | 372 | 2471 | 22.7 |  |
|  | #09 | 245 | 293 | 1668 | 407 | 2613 | 23.3 |  |
|  | #10 | 312 | 350 | 1861 | 474 | 2997 | 25.3 |  |
|  | **Total** | **1868** | **2216** | **12707** | **3098** | **19889** | **23.2** | **1.4** |
|  |  |  |  |  |  |  |  |  |
| Male | #01 | 32 | 21 | 48 | 64 | 165 | 32.1 |  |
|  | #02 | 74 | 71 | 147 | 163 | 455 | 31.9 |  |
|  | #03 | 34 | 50 | 118 | 84 | 286 | 29.4 |  |
|  | #04 | 45 | 49 | 120 | 130 | 344 | 27.3 |  |
|  | #05 | 73 | 75 | 218 | 224 | 590 | 25.1 |  |
|  | #06 | 120 | 111 | 278 | 310 | 819 | 28.2 |  |
|  | #07 | 49 | 41 | 84 | 121 | 295 | 30.5 |  |
|  | #08 | 39 | 32 | 93 | 101 | 265 | 26.8 |  |
|  | #09 | 40 | 42 | 121 | 148 | 351 | 23.4 |  |
|  | **Total** | **506** | **492** | **1227** | **1345** | **3570** | **28.0** | **3.0** |
| ^1^ Transmission of the meiotic recombination tester through maternal (female), paternal (male) or both gametes (selfed) determined by reciprocal crosses (female, male) or self-pollination. | | | | | | | | |
|  |  |  |  |  |  |  |  |  |
| ^2^ Seeds showing both red and green fluorescence. | | | | | | | | |
| ^3^ S.D. - standard deviation, calculated from the individual crosses/self-pollinations | | | | | |  |  |  |
