# Supplementary material for: Correction: Polyploidization increases meiotic recombination frequency in Arabidopsis
Source: BMC Biol. 2012 Apr 18;10:33. doi: 10.1186/1741-7007-10-33 (PMC3361494; doi:10.1186/1741-7007-10-33)
Supplement: Additional file 3 — Additional Table 3. [file 1741-7007-10-33-S3.DOC]

| **Additional Table 3** | | | | | | | | |
| --- | --- | --- | --- | --- | --- | --- | --- | --- |
| **Meiotic recombination frequencies (MRF) in allotetraploid *A. suecica* with single copy meiotic tester** | | | | | | | | |
| **Meiosis^1^** | **Plant ID** | **Seed fluorescence** | | | | **Seeds total** | **MRF (%)** | **S.D.^3^ (%)** |
|  |  | **Green-only** | **Red-only** | **Yellow^2^** | **None** |  |  |  |
| Female | #01 | 31 | 33 | 283 | 268 | 615 | 10.4 |  |
|  | #02 | 45 | 53 | 268 | 304 | 670 | 14.6 |  |
|  | #03 | 25 | 31 | 262 | 236 | 554 | 10.1 |  |
|  | #06 | 49 | 44 | 263 | 240 | 596 | 15.6 |  |
|  | #07 | 31 | 53 | 272 | 257 | 613 | 13.7 |  |
|  | **Total** | **181** | **214** | **1348** | **1305** | **3048** | **13.0** | **2.5** |
|  |  |  |  |  |  |  |  |  |
| Selfing | #01 | 59 | 61 | 315 | 79 | 514 | 27.0 |  |
|  | #02 | 35 | 44 | 207 | 52 | 338 | 27.0 |  |
|  | #03 | 30 | 31 | 186 | 40 | 287 | 24.2 |  |
|  | #06 | 65 | 68 | 320 | 53 | 506 | 31.1 |  |
|  | #07 | 86 | 94 | 456 | 96 | 732 | 28.7 |  |
|  | **Total** | **275** | **298** | **1484** | **320** | **2377** | **28.0** | **2.6** |
|  |  |  |  |  |  |  |  |  |
| Male | #01 | 121 | 117 | 227 | 256 | 721 | 33.0 |  |
|  | #02 | 151 | 147 | 314 | 355 | 967 | 30.8 |  |
|  | #03 | 53 | 67 | 211 | 156 | 487 | 24.6 |  |
|  | #06 | 122 | 111 | 285 | 247 | 765 | 30.5 |  |
|  | #07 | 151 | 157 | 375 | 396 | 1079 | 28.5 |  |
|  | **Total** | **598** | **599** | **1412** | **1410** | **4019** | **29.8** | **3.1** |
| ^1^ Transmission of the meiotic recombination tester through maternal (female), paternal (male) or both gametes (selfed) determined by reciprocal crosses (female, male) or self-pollination | | | | | | | | |
|  |  |  |  |  |  |  |  |  |
| ^2^ Seeds showing both red and green fluorescence | | | | | | | | |
| ^3^ S.D. - standard deviation, calculated from the individual crosses/self-pollinations | | | | | |  |  |  |
